# Hybrid sequencing resolves two germline ultra-complex chromosomal rearrangements consisting of 137 breakpoint junctions in a single carrier

**DOI:** 10.1007/s00439-020-02242-3

**Published:** 2020-12-14

**Authors:** Jesper Eisfeldt, Maria Pettersson, Anna Petri, Daniel Nilsson, Lars Feuk, Anna Lindstrand

**Affiliations:** 1 Department of Molecular Medicine and Surgery, Karolinska Institutet, Karolinska University Hospital Solna, 171 76 Stockholm, Sweden; 2 Department of Clinical Genetics, Karolinska University Hospital, Stockholm, Sweden; 3 Science for Life Laboratory, Karolinska Institutet Science Park, Solna, Sweden; 4 Science for Life Laboratory Uppsala, Department of Immunology, Genetics and Pathology, Uppsala University, Uppsala, Sweden

## Abstract

Chromoanagenesis is a genomic event responsible for the formation of complex structural chromosomal rearrangements (CCRs). Germline chromoanagenesis is rare and the majority of reported cases are associated with an affected phenotype. Here, we report a healthy female carrying two de novo CCRs involving chromosomes 4, 19, 21 and X and chromosomes 7 and 11, respectively, with a total of 137 breakpoint junctions (BPJs). We characterized the CCRs using a hybrid-sequencing approach, combining short-read sequencing, nanopore sequencing, and optical mapping. The results were validated using multiple cytogenetic methods, including fluorescence in situ hybridization, spectral karyotyping, and Sanger sequencing. We identified 137 BPJs, which to our knowledge is the highest number of reported breakpoint junctions in germline chromoanagenesis. We also performed a statistical assessment of the positioning of the breakpoints, revealing a significant enrichment of BPJ-affecting genes (96 intragenic BPJs, 26 genes, *p* < 0.0001), indicating that the CCRs formed during active transcription of these genes. In addition, we find that the DNA fragments are unevenly and non-randomly distributed across the derivative chromosomes indicating a multistep process of scattering and re-joining of DNA fragments. In summary, we report a new maximum number of BPJs (137) in germline chromoanagenesis. We also show that a hybrid sequencing approach is necessary for the correct characterization of complex CCRs. Through in-depth statistical assessment, it was found that the CCRs most likely was formed through an event resembling chromoplexy—a catastrophic event caused by erroneous transcription factor binding.

**Electronic supplementary material:**

The online version of this article (10.1007/s00439-020-02242-3) contains supplementary material, which is available to authorized users.

## Background

Complex chromosomal rearrangements (CCRs) are structural variants (SVs) consisting of multiple adjacent breakpoint junctions (BPJs). The vast majority of CCRs are reported in cancers, and most of the current knowledge on CCRs originate from such studies (Collins et al. 2017). However, a growing number of CCRs are reported also in the germline (Collins et al. 2019), and such CCRs are known to cause a variety of disorders, including intellectual disability and dysmorphism (Eisfeldt et al. 2019). CCRs arise through a multitude of events, including breakage-fusion-bridge (BFB) cycles (McClintock 1941), chromoanasynthesis (Liu et al. 2011), chromothripsis (Zhang et al. 2013), and chromoplexy (Baca et al. 2013).

BFB cycles may be initiated through telomeric dysfunction. Chromatids carrying dysfunctional telomeres may undergo rearrangements and fuse with other chromatids. During anaphase, these fused chromatids will be torn apart, resulting in rearranged chromatids that are lacking the telomere. Since the resulting chromatids have lost the telomere, they may fuse and be torn apart the next cell cycle, allowing for continuous cycles of breakage, fusion and bridging (McClintock 1941).

CCRs formed through BFB cycles are, therefore, mainly terminal rearrangements, they include duplications, deletions as well as copy number neutral fragments (Zakov and Bafna 2015). CCRs formed through BFB cycles may also be recognized based on the characteristic, non-random orientation of the DNA fragments involved, and various algorithms are described for such purposes (Kinsella and Bafna 2012).

In contrast to BFB cycles, chromothripsis is a single event of localized scattering of one or a few (< 4) chromosomes; once scattered, the fragments are rapidly joined in a seemingly random fashion (Pellestor 2019). Chromothripsis may involve deletions and copy number neutral fragments (Korbel and Campbell 2013) and may be initiated through numerous events, including the formation of micronuclei, viral insertion, or radiation (Koltsova et al. 2019). Chromothripsis has been observed in cancer (Cortés-Ciriano et al. 2020) as well as in germline (Macera et al. 2015).

Chromoanasynthesis is a catastrophic event, occurring during DNA replication and may be initiated by endogenous factors, including DNA secondary structures, as well as exogenous factors, including radiation, causing the DNA polymerase to replicate the DNA in an aberrant way (Liu et al. 2011). Chromoanasynthesis is characterized by non-clustered breakpoints, copy number states including both deletions and duplications, and templated insertions in the BPJs (Korbel and Campbell 2013; Zepeda-Mendoza and Morton 2019).

Chromoplexy is a recently discovered mechanism of CCR formation (Baca et al. 2013) which usually involves several chromosomes (> 2), and is generally a copy number neutral event (Pellestor 2019). Chromoplexy is believed to occur due to aberrant binding of transcription factors (Haffner et al. 2010). As such, the BPJs will cluster within genes, and may involve multiple co-transcribed genes. Chromoplexy has so far only been observed in cancers (Zhang et al. 2013), and with only a few cases reported.

In aggregate, these events involve different parts of the genetic repair machinery, and occur due to catastrophic errors in the most fundamental activities of the cell, including cell division, DNA replication and transcription.

As such, there is a great value in characterizing CCRs, the molecular characterization of CCRs may provide insights on genetic repair mechanisms and double-stranded breakage (Koltsova et al. 2019), as well as provide details on the structure and function of the genome in general (Pellestor 2019).

However, the correct characterization of CCRs is a difficult task, and commonly involves a large number of experiments, as well as time-consuming and manual analyses. Today, whole-genome sequencing (WGS) is the main method of choice for solving CCRs, and a variety of WGS methods have been applied to solve CCRs, including short-read sequencing (Nazaryan-Petersen et al. 2018), nanopore sequencing (Stancu et al. 2017), linked short-read WGS (Ott et al. 2018), and optical mapping (Chan et al. 2018). Given the novelty of these technologies, and the rarity of the CCR events, the choice of WGS method can be a great challenge. It is clear that each technology comes with a variety of advantages and disadvantages (Eisfeldt et al. 2019) and that the data may be analyzed through a diversity of bioinformatic pipelines (Stancu et al. 2017).

Here, we present a female with no reported health issues except for fertility problems carrying two distinct germline de novo chromosomal rearrangements involving a total of 6 chromosomes and 137 breakpoints, which is, to our knowledge, the largest number of reported BPJs in a germline CCR so far. The rearrangements [(*t*(X;21;19;4) and *t*(7;11)] were characterized using Illumina short-read WGS, Oxford nanopore WGS, 10X Genomics Chromium WGS, as well as Bionano optical mapping. We have compared the results of each method, and we find (1) that the methods are complementary, and (2) that a hybrid-sequencing approach is necessary for the complete characterization of these CCRs. Lastly, we provide a detailed description of the two CCRs, showing that they were formed as two separate events and through different cellular mechanisms: while *t*(7;11) was most likely formed through a replicative mechanism, *t*(X;4;19;21) was most likely formed through chromoplexy.

## Results

### Cytogenetic analyses

**Fig. 1:**
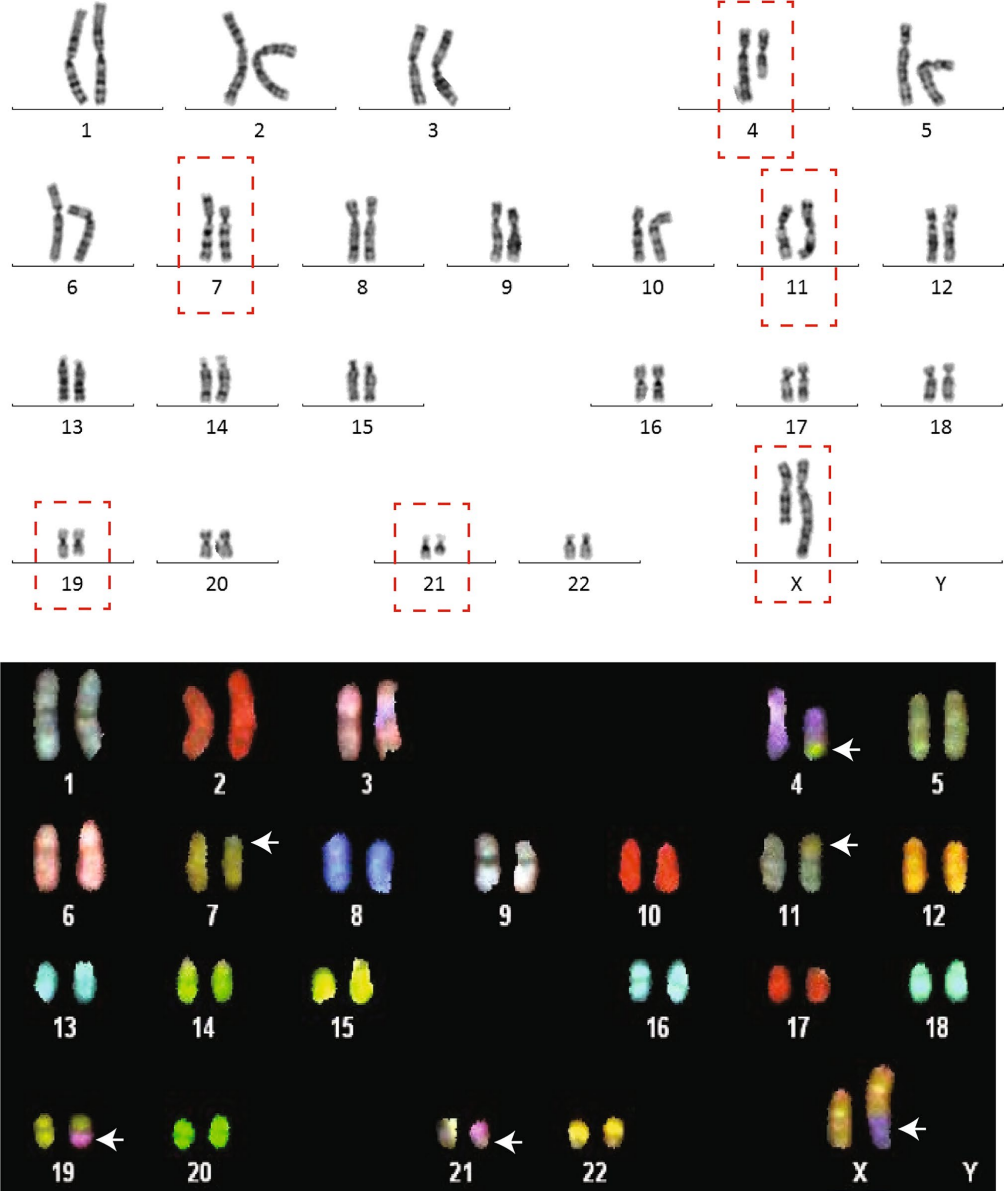
Chromosome analysis reveals complex translocations involving a total of six chromosomes. The karyotype of the patient was set as 46,X,t(X;21;19;4)(q26;q21;q13;q21)t(7;11)(p13;p15) after initial chromosome analysis (upper figure). Follow-up analysis with spectral karyotyping (SKY) was performed to visualize the chromosomal segments that had been rearranged, especially the chromosome 19 and 21 segments that were not distinguishable from the karyotype. White arrows indicate the different segments (lower figure)

Chromosome analysis revealed two seemingly balanced de novo translocations involving chromosomes 4, 19, 21 and X and chromosomes 7 and 11, respectively, and the initial karyotype was 46,X,*t*(X;21;19;4)(q26;q21;q13;q21)*t*(7;11)(p13;p15) (Fig. 1).

### Whole-genome sequencing analyses

**Fig. 2:**
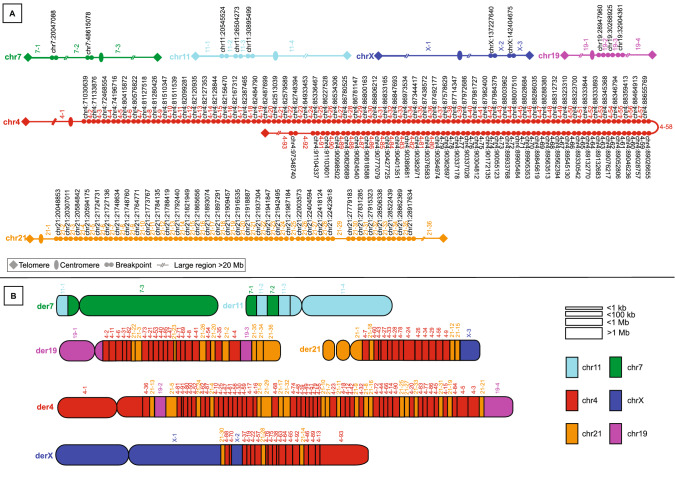
Detailed maps of the *t*(7;11) and *t*(X;21;19;4) rearrangements. **a** The breakpoint positions on chromosome 4, 19, 21, and X. **b** A diagram detailing the structure of *t*(X;21;19;4). Each block represents a fragment involved in the CCR, and the coloring illustrates the chromosome of origin of each fragment (turquoise: chromosome 11, green: chromosome 7, purple: chromosome 19, red: chromosome 4, orange: chromosome 21, and dark blue: chromosome X). The exact positioning and orientation of the fragments are presented in Supplementary Table 1

**Fig. 3:**
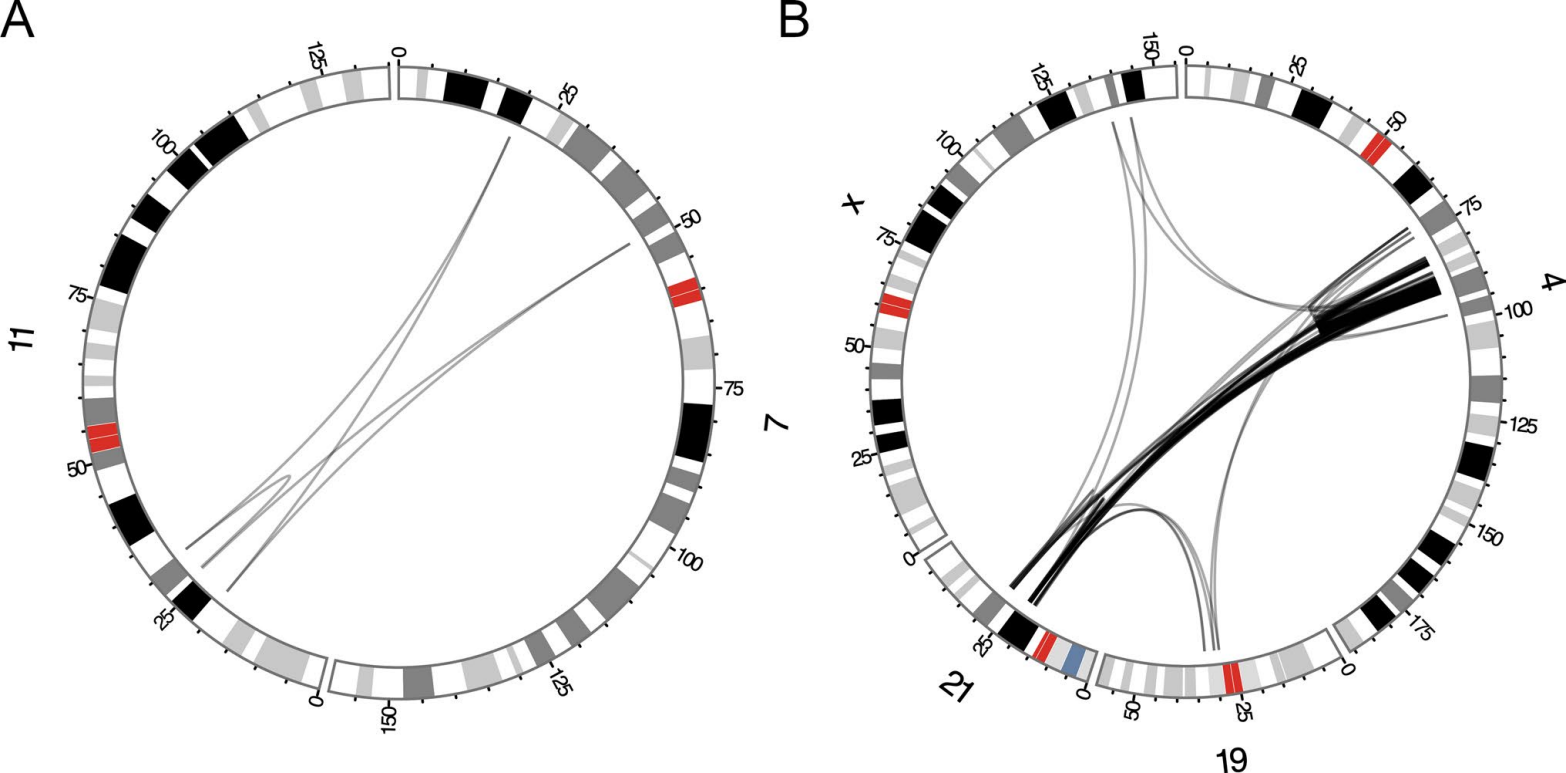
Circos plots illustrating the positioning of the BPJs involved in the complex rearrangement. The black arcs indicate the BPJs of **a** the *t*(7;11) rearrangement, and **b** the BPJs of the *t*(X;21;19;4) rearrangement

WGS analysis including short-read paired end (PE) WGS, linked-read WGS, nanopore WGS and Bionano optical mapping revealed that the rearrangement was ultra-complex and identified a total of 137 BPJs. The two rearrangements were confirmed to be completely separated and the *t*(7;11) translocation was far less complex than the *t*(X;21;19;4) rearrangement, involving 5 and 132 breakpoints, respectively (Figs. 2, 3). The breakpoints were confined to single-chromosome arms on all chromosomes involved and chromosome 4 and chromosome 21 were the most shattered chromosomes with 92 and 35 breakpoints, respectively (Figs. 2, 3). No additional chromosomes except those previously detected by karyotyping were involved in the CCRs.

Genomic segments involved in the rearrangement, apart from the end segments (i.e., chromosomal segments including the telomere), ranged from 10 s of megabases to less than 100 bp (Supplementary Table 1). Considering the high number of fragments, the rearrangement was remarkably balanced, involving 51 deletions totaling 100 Kbp (Supplementary Table 1). Three of the five breakpoints in the *t*(7;11) rearrangement were balanced and two breakpoints had deletions of one and two nucleotides, respectively. In the *t*(X;21;19;4) rearrangement, 84/132 (64%) breakpoints harbored deletions (median size = 80 bp, min = 1 bp, max = 26 Kbp, Supplementary Table 1). No duplications were detected. A total of 23 protein-coding genes were disrupted by one (*n* = 9) or several (*n* = 14) breakpoints (Table 1). Two of those genes were associated with human disease (*ANO3* associated with Dystonia 24, OMIM 610110, and *ABCG2* associated with Gout susceptibility, OMIM 138900) (Table 1). Except for *RASGEF1B*, all the affected protein-coding genes had a pLI score (Lek et al. 2016) lower than what is expected of genes causing Mendelian disease (0.9) (Table 1).

**Table 1:** Genes affected by the complex rearrangements

Gene	Number of BPJs	Chr	Start	End	Transcriptional strand	MIM phenotype	pLI
*t*(7;11)							
***ABCA13***	2	7	48211057	48687091	+		8.8e^−114^
***ANO3***	2	11	26210670	26684836	+	610110	1.2e^−17^
***DCDC5***	2	11	30885150	31014233	−		4.7e^−8^
*LOC101927668*	2	7	19958604	20180049	−		N.a.
*t*(X;21;19;4)							
***ARHGAP24***	14	4	86396284	86923823	+		3.9e^−10^
***ABCG2***	12	4	89011416	89152474	−	614490, 138900	2.1e^−32^
***AFF1***	12	4	87856154	88062206	+		0.71
***FAM13A***	8	4	89647105	89978346	−		1.9e^−18^
***PRKG2***	8	4	82008524	82136271	−		0.65
***C4orf22***	6	4	81256874	81884910	+		2.1e^−9^
*CYYR1*-*AS1*	6	21	27765954	27941571	+		N.a.
***HERC3***	6	4	89513574	89629693	+		0.07
***MMRN1***	6	4	90816052	90875780	+		2.1e^−25^
***NCAM2***	6	21	22370633	22914517	+		0.23
***NUDT9***	6	4	88343728	88380606	+		9.2e^−6^
***CCSER1***	4	4	91048684	92523370	+		0.000789
***HSD17B11***	4	4	88257674	88312455	−		2.1e^−7^
*LOC101928978*	4	4	84889235	85220322	−		N.a.
***PTPN13***	4	4	87515468	87736329	+		1.4e^−25^
***RASGEF1B***	4	4	82347547	82393082	−		0.92
***MAPK10***	3	4	86933449	87374283	−		0.28
***CYYR1***	2	21	27838528	27945723	−		0.0004
***DPY19L3***	2	19	32896655	32976799	+		2.9e^−7^
***GPRIN3***	2	4	90165429	90229161	−		4.1e^−13^
***HERC6***	2	4	89299891	89364249	+		1.5e^−14^
*LINC00989*	2	4	80413747	80497614	+		N.a.
*LOC100420587*	2	19	28926295	29218601	−		N.a.
*LOC101928942*	2	4	82086094	82114549	+		N.a.
*MIR548*X*HG*	2	21	19933583	20132130	−		N.a.
***PRR27***	2	4	71019904	71032326	+		0.0002

Protein-coding genes in bold

*BPJ* breakpoint junction, *N.a.* not applicable

A total of 23 fusions of genes (disrupted transcripts positioned next to each other due to the rearrangement) were formed (Supplementary Table 2, Supplementary Table 1). However, these fused genes lack promoter regions, or are fused in incompatible orientations, and are therefore unlikely to be transcribed into a stable RNA.

Next, we analyzed topologically associated domains (TADs) using a publicly available Hi-C dataset (Lajoie et al. 2015), produced from cerebellar astrocytes (Table 2). In total, 28 TADs are affected in this dataset involving all rearranged chromosomes in our patient, 5 by breakpoints from the *t*(7;11) rearrangement, and 23 by breakpoints from the *t*(X;21;19;4) rearrangement (Table 2). The breakpoints are unevenly spread across the TADs, and the majority of the breakpoints (156) cluster within the 5 most affected TADs (tad7, tad92, tad90, tad89, tad83). Notably, 127 BPJ were found to bridge across TADs (Supplementary Table 1). Four breakpoints were not located in any TAD and were, therefore, excluded from this analysis.

**Table 2:** TADs affected by the complex rearrangement

TAD	Chromosome	Start	End	Length	BPJ	Inter-TAD BPJ	BP	Protein-coding genes	MIM morbid genes
tad71	chr4	70560001	71440000	879	4	4	4	17	2
tad72	chr4	71480001	72600000	1119	2	2	2	8	2
tad75	chr4	74040001	75080000	1039	2	2	2	15	2
tad82	chr4	79920001	81240000	1319	6	6	6	5	3
tad83	chr4	81280001	82280000	999	17	16	18	3	0
tad84	chr4	82320001	83240000	919	13	12	14	1	0
tad86	chr4	84520001	85480000	959	5	4	6	2	0
tad88	chr4	85960001	87240000	1279	16	14	18	2	0
tad89	chr4	87280001	88200000	919	22	22	22	6	0
tad90	chr4	88240001	89480000	1239	32	28	36	16	6
tad91	chr4	89520001	90040000	519	13	12	14	4	0
tad92	chr4	90080001	91240000	1159	33	28	38	4	1
tad23	chr7	19800001	20720000	919	2	2	2	4	0
tad59	chr7	48240001	49120000	879	2	2	2	1	0
tad20	chr11	20520001	21480000	959	2	2	2	3	1
tad27	chr11	26320001	26640000	319	2	2	2	2	1
tad32	chr11	30280001	31080000	799	2	2	2	3	0
tad30	chr19	28080001	29320000	1239	2	2	2	0	0
tad32	chr19	30080001	31200000	1119	2	2	2	6	1
tad35	chr19	32880001	32960000	79	2	2	2	1	0
tad6	chr21	19800001	20680000	879	8	8	8	0	0
tad7	chr21	20720001	22360000	1639	39	36	42	0	0
tad8	chr21	22400001	23360000	959	6	6	6	1	0
tad13	chr21	27000001	28160000	1159	6	6	6	5	2
tad14	chr21	28200001	29080000	879	6	4	8	2	0
tad137	chrX	137200001	137840000	639	2	2	2	1	0
tad141	chrX	141320001	142560000	1239	2	2	2	1	0

*TAD* topologically associated domain, *BPJ* breakpoint junction, *BP* breakpoint, *MIM* Mendelian Inheritance in Man

The affected TADs contain a total of 113 genes, of which 21 are known MIM morbid genes (Supplementary Table 1). Eleven of those follow an autosomal-dominant inheritance pattern and span a wide range of phenotypes, including three genes linked to various dental issues (AMTN, *ENAM, DSPP*) (Nakayama et al. 2015; Dong et al. 2000; Crosby et al. 1995) and *APP* known to cause Alzheimer disease when duplicated (Adler et al. 1991). Eight genes have a pLI score > 0.9, including one MIM morbid gene (*SLC4A4*) linked to autosomal recessive disease (Table 2, Supplementary Table 1). The genes located within affected TADs were further analyzed with a PANTHER GO biological process statistical overrepresentation test. This test revealed a total of 32 significantly enriched biological processes (*P* < 0.05, false discovery rate < 0.05) (Supplementary Table 1).

Analyzing the BPJs on the nucleotide level revealed distinct mutational signatures for the two separate complex rearrangements. The *t*(7;11) rearrangement showed rare single-nucleotide variants (SNVs), microhomology (4 bp) and a templated insertion in the junctions (Supplementary Fig. 1, Supplementary Table 1), consistent with replicative errors such as fork-stalling and template-switching (FoSTeS)/microhomology-mediated break-induced replication (MMBIR) (Weckselblatt and Rudd 2015) that are typical features of chromoanasynthesis. In contrast, the *t*(X;21;19;4) rearrangement consistently showed blunt ends, little to no microhomology, non-templated insertions and small deletions in the junctions (Table 2, Supplementary Fig. 1), consistent with non-homologous end-joining (NHEJ) (Weckselblatt and Rudd 2015). Genomic segments as small as 33 bp have previously been shown to sometimes be processed by the repair machinery during reassembly of shattered chromosomes (Slamova et al. 2018), so the large insertions were first manually checked and then checked using BLAT, which generated no specific matches. Next, we used RepeatMasker (Smit et al. 2013) to analyze all insertions larger than 50 bp, revealing that these insertions contain large amounts of simple repeats (29% vs 1.5% in GRCh38 (Smit et al. 2013)). Interestingly, one insertion was found to be a chimera of an SVA and srpRNA (Supplementary Dataset 1).

Out of the 137 breakpoints, 51 (37%) were completely balanced and 45 (33%) had losses of less than 100 nucleotides (Table 3, Supplementary Table 1). Among the total 137 BPJs, 44 (32%) showed microhomology in the junctions, but no more than 4 nucleotides (Table 3, Supplementary Fig. 1, Supplementary Table 1). A total of 53 junctions (39%) had insertions, of which one (1%) seemed templated from nearby sequences.

**Table 3:** Breakpoint junction (BPJ) characteristics

*t*(7;11)			
By breakpoint	Total number	5	100.0%
	Balanced	3	60%
	<10 nt deletion	2	40%
By junction	Total number	5	100%
	Microhomology, total	2	40%
	< 2 nt	1	20%
	2–10 nt	1	20%
	Insertions, total	1	20%
	< 2 nt	0	0%
	< 20 nt	0	0%
	< 100 nt	1	20%
	> 100 nt	0	0%
*t*(X;21;19;4)			
By breakpoint	Total number	132	100%
	Balanced	48	36%
	< 10 nt deletion	15	11%
	< 100 nt deletion	29	22%
	< 1000 nt deletion	18	13%
	< 10,000 nt deletion	21	16%
	> 10,000 nt deletion	2	2%
By junction	Total number	132	100.0%
	Microhomology, total	39	30%
	< 2 nt	18	14%
	2–10 nt	21	16%
	Insertions, total	52	39%
	< 2 nt	7	5%
	< 20 nt	14	11%
	< 100 nt	22	16%
	> 100 nt	9	7%

*nt* nucleotide

Parental origin of both de novo rearrangements could not be determined as no parental samples were available for analysis.

### Statistical assessment of the derivative chromosome structure and breakpoint junction characteristics of the *t*(X;21;19;4) rearrangement

The *t*(7;11) rearrangement carries features typical to chromoanasynthesis including dispersed interchromosomal translocations, as well as traces of replicative repair mechanisms (Zepeda-Mendoza and Morton 2019). In contrast, an in-depth statistical analysis was required to determine the mechanism of formation of the *t*(X;21;19;4) CCR. As noted previously, the *t*(X;21;19;4) CCR BPJ carries signatures consistent with NHEJ, which is found in a diversity of mechanisms of CCR formation, including chromothripsis, chromoplexy (Pellestor 2019), and BFB cycles (Marotta et al. 2013).

Analyzing the distribution of DNA fragments across the derivative chromosomes, we find that the fragments are spread across the derivative chromosomes in a non-random fashion. In particular, we note that derivative chromosome 4 contain 48% (*n* = 45, *p* = 10^−6^) of the fragments originating from chromosome 4, and 56% (*n* = 20, *p* = 10^−4^) of the fragments originating from chromosome 21 (Table 4). Conversely the derivative chromosome 21 is depleted in fragments originating from chromosome 4 (*n* = 13, *p* = 0.007), and chromosome 21 (*n* = 4, *p* = 0.034). In addition, there is an absence of fragment exchange between some of the derivative chromosome, including chromosome 19 and X. These results indicate that the *t*(X;21;19;4) CCR was formed either through a non-random process, causing skewed re-assembly of the chromosomes, or that the *t*(X;21;19;4) CCR was formed through a progressive multistep process, allowing the fragments to be reused in a directed, non-random fashion.

**Table 4:** Analysis of the distribution of aberrant fragments in *t*(X;21;19;4)

Derivative chromosome	Chromosome of origin Fraction of aberrant fragments							
	4		19		21		X	
	Fraction	*P* value	Fraction	*P* value	Fraction	*P* value	Fraction	*P* value
Der(4)	0.48	< 0.001	0.5	0.28	0.56	< 0.001	0	0.37
Der(19)	0.19	0.12	0.5	0.28	0.25	0.5	0	0.37
Der(21)	0.14	0.01	0	0.28	0.11	0.03	0.33	0.37
Der(X)	0.18	0.08	0	0.28	0.08	0.02	0.67	0.16

Number of fragments in each derivative chromosome			
Der(4)	Der(19)	Der(21)	Der(X)
67	28	18	29

Analyzing the orientation of the fragments, we found no statistically significant patterns, and inverted and non-inverted fragment fusions appear equally likely (Table 5). Such patterns are consistent with a number of mechanisms, including chromoplexy and chromothripsis (Korbel and Campbell 2013), but is inconsistent with BFB cycles, a mechanism known to produce CCRs enriched in *head*-to-*head* and *tail*-*to*-*tail* fusions (Kinsella and Bafna 2012).

**Table 5:** Orientation of the fused fragments in t(X;21;19;4)

Derivative chromosome	Tail-to-head		Head-to-tail		Head-to-head		Tail-to-tail	
	Count	*P* value	Count	*P* value	Count	*P* value	Count	*P* value
Der(4)	19	0.29	15	0.39	16	0.5	16	0.5
Der(19)	3	0.06	9	0.26	8	0.41	8	0.41
Der(21)	4	0.45	5	0.44	4	0.45	4	0.45
Der(X)	6	0.45	7	0.26	4	0.35	4	0.35
Overall	32	0.46	36	0.31	32	0.46	32	0.46

96 (72%) of the *t*(X;21;19;4) BPJs affect a total of 26 genes. These genes are spread across chromosomes 4, 19 and 21 (Table 1). The BPJs cluster within these genes; for instance, 14 BPJs were located within *ARHGAP24,* and 16 genes are affected by 4 or more BPJs. Applying Monte Carlo methods, we find that the *t*(X;21;19;4) is enriched in intragenic BPJs (*p* < 0.0001). In addition, although the breakpoints cluster within genes, the genes themselves are not clustered: in total, these genes span roughly 34 Mbp, with 22 Mbp, 4 Mbp, and 8 Mbp distributed on chromosome 4, 19, and 21, respectively.

To investigate the expression pattern of these affected genes, a GTex multigene query (https://gtexportal.org/) was performed on the 15 protein-coding genes affected by at least 3 breakpoints, revealing that all of those genes are co-expressed in testis, but no other tissue (Supplementary Fig. 2).

### Comparison of the sequencing technologies

The two rearrangements were characterized using a combination of four technologies: Illumina short-read sequencing, linked short-read sequencing, nanopore sequencing, and optical mapping. The *t*(7;11) consists of five BPJs, all of which were detected by all four technologies (Supplementary Table 1).

In contrast, none of the technologies were able to detect all of the 132 BPJs in the *t*(X;21;19;4) rearrangement (Table 6). Nanopore sequencing detected the largest number of the *t*(X;21;19;4) BPJs (*n* = 120, 90.9%), and produced the largest number of total calls as well (14,730). Illumina PE sequencing detected the second largest amount of the *t*(X;21;19;4) BPJs (*n* = 119, 90.2%), and produced a relatively small number of calls (5084).

**Table 6:** The number of detected chromosomal breakpoints in *t*(X;21;19;4) with the different sequencing technologies

	Illumina PE (TIDDIT)	Nanopore (sniffles)	Optical mapping (bionano access)	Linked reads (Longranger)
Illumina PE (TIDDIT)	119 (5084)	–	–	–
Nanopore (sniffles)	130	120 (14,730)	–	–
Optical mapping (bionano access)	119	120	19 (12,057)	–
10 × chromium (Longranger)	130	124	88	87 (1983)

The table presents the number of gold-standard breakpoints detected for each separate technology, as well as each pairwise combination. The numbers within parenthesis illustrate the total number of calls

**Fig. 4:**
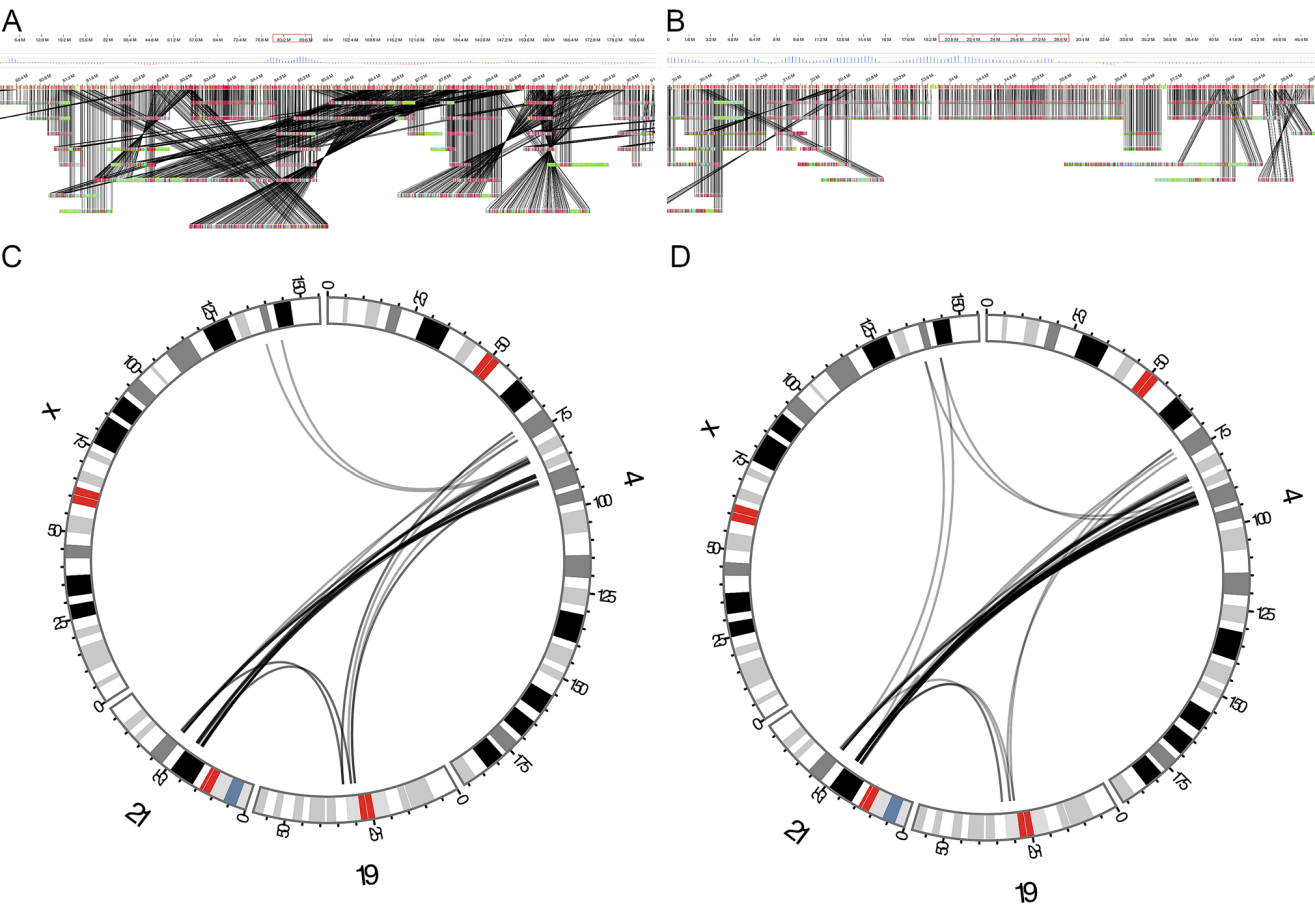
Manual inspection of the Optical mapping data. **a** A screenshot of chromosome 4, using Bionano Access. **b** A screenshot of chromosome 21, using Bionano Access. **c** A Circos plot presenting all Bionano access translocation calls involving chromosomes 4, 21, 19 and X. **d** A Circos plot presenting the interchromosomal BPJs involving chromosomes 4, 21, 19 and X

Optical mapping detected the lowest amount of the *t*(X;21;19;4) BPJs (*n* = 19, 14.4%), and produced the second largest number of calls (12,057). Analyzing the rearrangement manually in Bionano Access, we do find a larger number of calls corresponding to the BPJs of *t*(X;21;19;4) (Fig. 4a–d) and comparing all interchromosomal SV Bionano *t*(X;21;19;4) calls (*n* = 33) to all confirmed interchromosomal SV BPJs (*n* = 56), it was found that Bionano produces a significant number of similar calls (Fig. 4c, d). However, only 22 of these calls are located within 100 Kbp of the verified breakpoint positions, indicating low resolution.

**Fig. 5:**
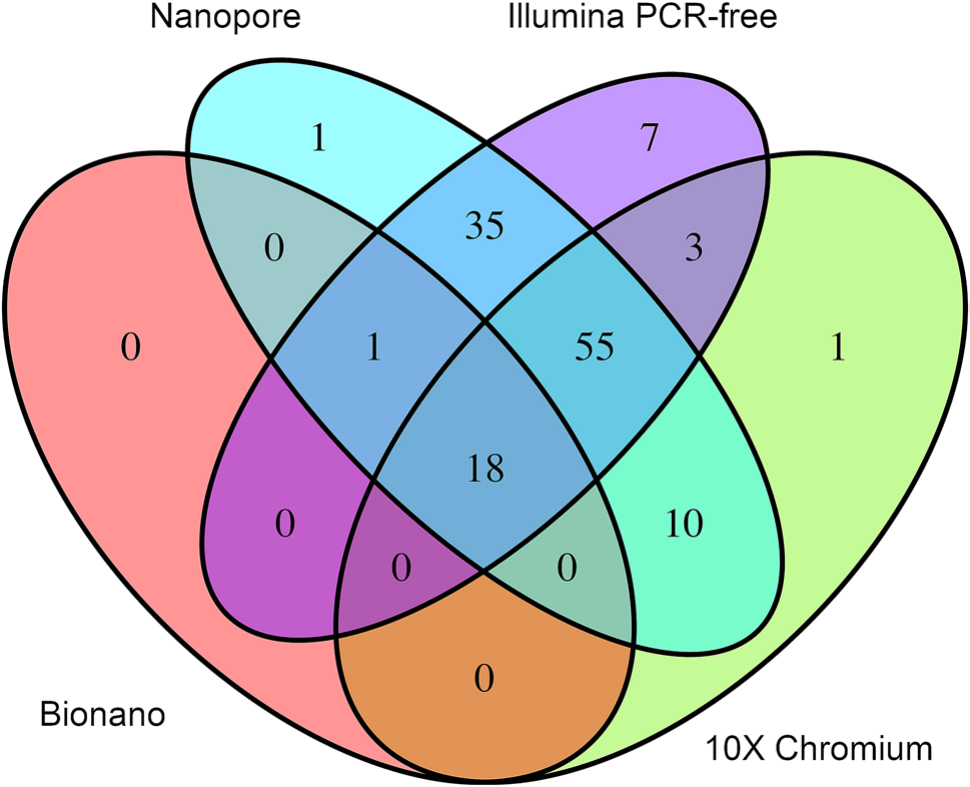
Comparison of the sequencing technologies. A Venn diagram illustrating the number of junctions detected by the four technologies in *t*(X;21;19;4)

Producing pairwise combinations of each technology, the detection rate is slightly increased. The highest detection rates were obtained by combining Illumina PE with either Nanopore WGS or 10 × Chromium WGS (Fig. 5, Table 6), allowing the automated detection of 130 BPJs (99%). Notably, there is only a small gain in adding a third-sequencing technology (Fig. 5). Inspecting the BPJs not detected by the various pipelines, it was found that the Illumina PE pipeline fails to detect BPJs in highly repetitive regions, as well as BPJs carrying large non-templated insertions (Supplementary Table 1). In contrast, nanopore WGS perform well in repetitive regions, but is limited by the high error rate and relatively low span-coverage. The linked short-read WGS is affected by various sequencing biases, such as GC content, and the sequencing coverage appears noisier than the standard Illumina PE, causing dropouts at the BPJ regions. Optical mapping appears ill-suited for these many and short fragments, and the usefulness of this technology is limited due to its low resolution.

### Hybrid de novo assembly of *t*(X;21;19;4)

We attempted to solve the CCR using a hybrid de novo assembly approach, potentially allowing automated characterization of the CCR. These hybrid assemblies were based on the Wtdgb2 (Ruan and Li 2020) assembly of the nanopore WGS data (Supplementary Fig. 3A), Supernova (Weisenfeld et al. 2017) assembly of the linked short-read WGS data (Supplementary Fig. 3B), and optical maps. Notably, only a few of the BPJs were assembled (Table 7), resulting in low detection rate compared to the mapping assembly approach (Table 6). The highest detection rate is obtained by performing a nanopore–Bionano hybrid assembly. However, with only 71 detected BPJs, the detection rate is clearly lower than most of the mapping-based approaches (Table 6).

**Table 7:** De novo assembly statistics summary in *t*(X;21;19;4)

	Detected BPJ	Calls	N50 (Mbp)	Size (Gbp)
Supernova (10 ×)	47	100,298	0.13	3.9
Wtdgb2 (nanopore)	68	2870	3.9	2.6
Quickmerge (10 × + nanopore)	1	6095	2	4.5
Bionano scaffold 1 (nanopore + Bionano)	71	3941	41	2.7
Bionano scaffold 2 (Bionano + Quickmerge)	28	118,984	45	2.8

Interestingly, Quickmerge removes the majority of the called BPJs (Table 7); however, it also removes the majority of calls present in the Supernova assembly. Quickmerge is, therefore, better suited for constructing reference genomes, as it removes noise and ambiguities at the cost of true variation.

Despite the low detection rate of these de novo assembly approaches, the resulting contigs were useful for validating the hypothesized derivative chromosomes. Contigs covering multiple aberrant fragments were especially useful, as such contigs allow for validation of the results obtained through mapping assembly (Supplementary Fig. 4).

Quality-wise, the best performance was achieved by combining the nanopore Wtdgb2 assembly with optical maps, yielding a relatively low number of calls (3941) indicating a tolerable amount of misassemblies while maintaining high contiguity and a relatively large assembly size (Supplementary Fig. 3C).

Even higher contiguity is obtained by merging the Supernova-WTDB2 Quickmerge results with optical mapping; however, the large number of calls indicate a large number of misassemblies (Supplementary Fig. 3D).

## Discussion

Herein, we utilized four high-throughput genomic technologies to fully resolve the structure of two CCRs that had arisen independently as de novo events in a healthy woman and found Illumina PE WGS and nanopore WGS to be the most promising hybrid-sequencing approach for solving CCRs consisting of a large number of small aberrant fragments. These two technologies offer high detection rate, and are orthogonal, hence variants detected by both methods may be considered validated.

Through a hybrid-sequencing approach and long read de novo assembly, the structure of the two CCRs was fully characterized. Subsequent breakpoint junction analysis enabled the most plausible mechanisms of formation to be determined. The *t*(7;11) rearrangement was determined to most likely have been formed through a replicative error mechanism, largely due to the templated insertions. Instead, an in-depth statistical analysis was performed to determine the mechanism of formation of *t*(X;21;19;4). Through these analyses, it was found that the CCR most likely was formed through a progressive multistep process, that the fragments were reinserted in random orientation, and that the breakpoints are widely spread across the genome but cluster within genes. All these signatures are consistent with chromoplexy, and the fact that the rearrangement is largely balanced, and spread across four chromosomes adds to this hypothesis (Pellestor 2019). A growing amount of literature is describing chromoplexy as a catastrophic event occurring during active transcription (Yi and Ju 2018), and as presented here the *t*(X;21;19;4) CCR, which is highly enriched in intragenic breakpoints would be consistent with this idea.

Our second-best hypothesis would be formation through chromothripsis, however, this hypothesis is likely to be rejected as chromothripsis is defined as one single localized cataclysmic event of breakage, followed by random reintegration of fragments (Korbel and Campbell 2013), which would be the opposite of the formation of *t*(X;21;19;4). The number of BPJs (132) in *t*(X;21;19;4) exceed the typical numbers reported in both chromoplexy and germline chromothripsis in the literature. We must note, however, that chromoplexy is a recently described mechanism of CCR formation, and that larger, more complex chromoplexy rearrangements are likely to be reported as they are detected.

In the *t*(X;21;19;4) CCR, insertions were found in 39% of the BPJs (Table 3) and 24 BPJs harbor repetitive non-templated insertions larger than 50 bp (Supplementary Table 1). In addition, most of the breakpoints (66%) contain deletions (Table 3). These findings provide clues on which cellular repair pathways underly the formation of the CCR. Alternative-NHEJ is a likely candidate, as it is known to produce large insertions and deletions at the BPJs (Seol et al. 2018) and has previously been described in chromoplexy (Zepeda-Mendoza and Morton 2019). In particular, polymerase theta-mediated end-joining (PTMEJ) is known to produce large insertions and these insertions are produced through fill-in synthesis that stabilizes the open ends (Black et al. 2016) and provides the homologous sequences used to join the open ends (Mateos-Gomez et al. 2017). Such DNA synthesis could explain the large random insertions observed at the BPJs of *t*(X;21;19;4) CCR. Furthermore, polymerase theta is known to include templated insertions at the BPJ (Schimmel et al. 2019) which can be seen at numerous BPJs, including BPJ 4–68 → 21–17 and 21–32 → 4–74 (Supplementary Figure S1).

Analyzing the co-expression pattern of the affected protein-coding genes, it was found that the affected genes are co-expressed in one tissue only, the testis (Figure S2). As *t*(X;21;19;4) is a germline rearrangement, formed through chromoplexy, these findings could imply that the rearrangement was formed during spermatogenesis. However, since we lack paternal DNA samples, this hypothesis cannot be tested. In particular, we would be interested in confirming whether the *t*(X;21;19;4) CCR is of paternal or maternal origin, as well as to check whether the CCR was formed before or after meiosis.

Single cell Hi-C studies of the various developmental stages of spermatozoa and ova could be utilized to overcome these issues. Through such analyses, 3D genomic maps could be constructed (https://github.com/lh3/hickit) (Nagano et al. 2017) and these maps could be used to assess the likelihood of a suggested mechanism of formation (Berthelot et al. 2015).

Comparing the four technologies used (Bionano optical mapping, Illumina PE WGS, linked-read WGS, and nanopore sequencing), we would recommend Illumina PE WGS as a first experiment for solving a CCR like the *t*(X;21;19;4) rearrangement presented here as it is the most cost-efficient approach, offering high detection rate, and a large selection of bioinformatic tools, at a relatively small cost. Follow-up experiments should be chosen based on the biological question, as well as the structure of the CCR.

Except for fertility problems, the proband is unaffected which is surprising given the high number of breakpoints. The reason, however, for the lack of a clinical phenotype is that the CCR largely consists of intragenic breakpoints disrupting no early-onset dominant disease genes (Table 1). In addition, the majority of disrupted genes are unlikely to be haploinsufficient (pLI < 0.9). The fact that 27 TADs containing a total of 113 genes were potentially affected by the CCRs (Table 2, Supplementary Table 1) is remarkable and highlights the importance of continued research into the clinical significance of TAD disruptions. We speculate that the TADs are perturbed so that the change of expression is not consistent with the gene-related disease. The majority of affected TADs and MIM morbid genes are found in the more complex *t*(X;21;19;4), which is likely to be formed through chromoplexy. As chromoplexy involves regions that are co-expressed and close in the 3D-space of the nucleus (Yi and Ju 2018), the 3D structure of the probands genome may, therefore, be less perturbed than what is shown in these one-dimensional TAD analyses (Table 2, Supplementary Table 1) which could also explain why the patient do not display a phenotype consistent with these disease genes. Alternatively, one could speculate that the rearrangement may cause late-onset disease, explaining why the proband currently do not display any disease phenotype, an example being *APP* (Supplementary Table 1), which is involved in Alzheimer disease (Adler et al. 1991), a well-known late-onset disease. Whether to test a healthy individual for late-onset disease or not is an ethical dilemma, however, both Hi-C and RNA-seq data would be useful in understanding the functional effect of such a complex rearrangement.

Despite fertility problems, the proband naturally conceived and gave birth to a healthy child carrying only the *t*(7;11) CCR. The fertility problems observed may be explained by a high number of gametes carrying unbalanced combinations of the two CCRs. Of the 64 gametes possible through alternate or adjacent segregation I (Supplementary Fig. 5), only 4 (6.25%) would carry a balanced set of chromosomes compared to 2/4 (50%) in carriers of reciprocal-balanced translocations (Morel et al. 2004).

## Conclusion

In conclusion, we present two de novo complex chromosomal rearrangements involving 6 chromosomes and 137 BPJs in a healthy female. The amount of BPJs are approximately twice as many as have been reported to this day in germline chromothripsis (Collins et al. 2017), and almost sixfold the amount of BPJs that have been reported in a healthy individual (De Pagter et al. 2015). Analysis of the BPJs suggests that a combination of repair machineries have operated in the same cell, and that the distinct rearrangements have occurred through different mechanisms. In this way, we illustrate that present day commercial genomic technologies are suitable for fully characterizing such a rearrangement, however, it is clear that a multi-omics approach is necessary for understanding its full structure and complexity.

The large amount of work and effort put into this single case illustrate how a single a patient can provide significant clues and insights to molecular mechanisms underlying these events; however, we also illustrate that personalized care may require costly and truly personalized analyses.

## Methods

### Clinical synopsis

The proband is a female who was first referred for genetic investigation at age 3 years because of short stature. The clinical investigation was dropped when the patient started catching up in height and she now reports as an adult as being of normal height compared to other women in her family, and otherwise healthy. She has a history of fertility problems likely due to the chromosomal rearrangement but has a healthy child conceived naturally who only inherited one translocation, the *t*(7;11).

### Cytogenetic analysis

Metaphase slides were prepared from peripheral blood cultures according to standardized protocols. Chromosome analysis was performed according to routine procedures with the GTG-banding technique and an approximate resolution of 550 bands per haploid genome was obtained.

### Short-read whole-genome sequencing

Genomic DNA derived from whole blood from the proband was sequenced at National Genomics Infrastructure (NGI), Stockholm, Sweden, using a PCR-free paired-end (PE) protocol; resulting in roughly 35X coverage. Data were processed and analyzed as described previously (Eisfeldt et al. 2019). Briefly, the data were pre-processed using the NGI-piper pipeline (https://github.com/NationalGenomicsInfrastructure/piper) and structural variants were called using the FindSV (https://github.com/J35P312/FindSV) pipeline that combines CNVnator (Abyzov et al. 2011) and TIDDIT (Eisfeldt et al. 2017). Variants of interest were visualized in Integrative Genomics Viewer (IGV) (Thorvaldsdóttir, Robinson, and Mesirov 2013).

### Linked-read whole-genome sequencing

Genomic DNA derived from whole blood from the proband was also sequenced using the 10 × Genomics Chromium WGS protocol and data were analyzed and processed as described previously (Eisfeldt et al. 2019). Data were analyzed using 10 × Genomics default pipelines Long Ranger V2.1.2 (https://support.10xgenomics.com/genome-exome/software/downloads/latest).

### Optical mapping

Optical mapping was performed on genomic DNA from the proband by running dual enzymes (BspQI, BssSI) on the Bionano Genomics (San Diego, CA, USA) Saphyr platform (https://bionanogenomics.com/support-page/saphyr-system). Analysis was performed as described previously (Eisfeldt et al. 2019). Briefly, the optical maps were analyzed using Bionano-solve (https://bionanogenomics.com/support-page/bionano-solve), aligned to Hg19 reference genome using Bionano RefAligner (version 5649) and output files were converted into VCF files using a custom script (https://github.com/J35P312/smap2vcf). Variants of interest were visualized in Bionano access.

### Nanopore whole-genome sequencing

Nanopore WGS was performed at National Genomics Infrastructure (NGI) Uppsala. The sequencing library was prepared using the LSK-109 ligation kit and sequencing was performed on the PromethION platform using the R9.4 flow cell. Bases were called using the Guppy base caller (https://nanoporetech.com) producing 2729,339 reads, with an average length of 15 Kbp, resulting in roughly 13 × coverage.

The resulting nanopore WGS data were aligned to Hg19 using Minimap2 (Li 2018), and variants were called using Sniffles (Sedlazeck et al. 2018), setting the minimum read support parameter to three reads. SV calls shorter than 2 Kbp were considered Indels (insertions/deletions) and were, therefore, removed.

### Statistical tests

Statistical tests were performed to describe the *t*(X;21;19;4) CCR, and to reject implausible mechanisms of formation. Binomial tests were performed to assess the randomness of the distribution of fragments among the derivative chromosomes, and to test for biases regarding the orientation of the aberrant fragments. The binomial tests were performed using the social statistics online binomial test calculator https://www.socscistatistics.com/tests/binomial/.

The tests for biased fragment orientation were run for each derivative chromosome, and each possible orientation (*Head*–*Head*, *Head*–*Tail*, *Tail*–*Head*, and *Tail*–*Tail*).

The number of trials were set to the number of fragments within the derivative chromosome being tested, and the probability of the outcome was set to 1/4 (i.e., the number of possible fragment orientations), and the number of observed occasions were set to the orientations as observed.

The randomness of the distribution of fragments was assessed by performing binomial tests for each derivative chromosome. Here, the number of trials were set to the number of fragments involved in *t*(X;21;19;4), the probability of the outcome was set to 1/4 (i.e., the number of chromosomes involved in *t*(X;21;19;4)). The number of observed occasions was set to the observed number of fragments within the derivative chromosome being analyzed.

A Monte Carlo test was performed to assess the enrichment of BPJs within genes.

We utilized the RefSeq gene annotation (assembly GCF_000001405.25) to count the number of intragenic *t*(X;21;19;4) BPJs. A BPJ was considered intragenic if any of its two breakpoints were located within a gene. Next, an equal number of random BPJs were simulated across chromosome 4, 19, 21 and X and the number of intragenic simulated BPJs were counted. The simulated BPJs were selected according to a random uniform distribution, such that all bases across chromosome 4, 19, 21 and X were equally likely to be affected by simulated rearrangements.

This procedure was repeated for 1000 iterations, and a *p* value was defined as the fraction of simulated CCRs carrying more intragenic BPJs than the observed number of intragenic BPJs in *t*(X;21;19;4).

### Breakpoint PCR and Sanger sequencing

Breakpoint PCR was performed on some BPJs that were too complex for WGS data analysis only. Primers were designed flanking the junctions approximately 500 bp away from the estimated breakpoint with M13 sequences attached. Breakpoint PCR was performed by standard methods using Phusion High-Fidelity DNA Polymerase (ThermoFisher Scientific, Waltham, MA, USA). Sanger sequencing of amplicons was performed according to standard protocols with M13 primers. Sequences were aligned using the BLAT tool (Kent 2002) and visualized in CodonCode Aligner (CodonCode Corp., Dedham, MA, USA).

### De novoassembly

De novo assembly was performed using Bionano optical mapping, nanopore long-read and linked short-read sequencing data. As mentioned previously, the Bionano optical maps were assembled using Bionano Solve. The nanopore WGS data were assembled using Wtdgb2 (Ruan and Li 2020), and the 10X Genomics Chromium linked reads were assembled using the Supernova assembler (Weisenfeld et al. 2017). The assemblies were aligned to Hg19 using Minimap2 (Li 2018), and SVs were called using Assemblatron (Eisfeldt et al. 2020).

A variety of hybrid assemblies were produced: the Wtdgb2 and Supernova assemblies were merged using the Quickmerge tool (Solares et al. 2018) and all previously mentioned assemblies were merged with Bionano optical maps using the Bionano Solve hybrid-scaffolding tool. The resulting hybrid scaffolds were aligned to Hg19 using Minimap2, and SVs were called using Assemblatron.

### Solving the complex chromosomal rearrangements

The BPJs were found by combining the filtered FindSV (Lindstrand et al. 2019) output with the filtered Sniffles (Sedlazeck et al. 2018) output using SVDB (Eisfeldt et al. 2017). Next, the BPJs were manually inspected through IGV (Thorvaldsdóttir et al. 2013), this inspection was necessary to resolve inaccuracies due to repeats or microhomology, as well as to find BPJs not called by any of the pipelines.

The filtered list of BPJs were manually compared the Optical mapping data, using Bionano Access, as well as the linked reads, using IGV and Loupe (https://support.10xgenomics.com). The manual comparison was also necessary due to the low resolution of these technologies, as well as the large amount of false-positive calls produced by the Bionano and Longranger pipelines. BPJs were considered true if they were supported by at least two of the WGS methods (either through calling or manual inspection), or by breakpoint PCR.

Aberrant DNA fragments were defined based on the quality controlled and filtered data. Except for the terminal fragments, each DNA fragment was associated with two BPJs, one at the head and one at the tail, forming chains of fragments, fused together through the BPJs. Lastly, the derivative chromosomes were defined using a custom script. This script “walks” through these chains, starting at a user-defined fragment (such as the first fragment of chromosome 4), and continues to the next fragment through the BPJs (or calls) associated with each fragment. This “walking” is continued until the script finds the terminal fragment in a chain and at that point the script ends and returns the path traveled through the chain of aberrant fragments. This path is a representation of the derivative chromosome, detailing the order and orientation of the fragments involved.

This chain was compared to the various sequencing data, de novo assemblies, as well as the cytogenetic results and was refined until the path was consistent with all signatures in each of the datasets.

### Comparison of sequencing technologies

The VCF files produced from each sequencing technology was compared to the confirmed BPJs of the *t*(X;21;19;4) CCR using SVDB query. The *t*(X;21;19;4) truth-set was prepared by converting the confirmed BPJ into a bedpe file. In addition, SVDB query was run using the following command:

*Svdb* –*query* –*query_vcf input.vcf* –*bedpedb truthset.bedpe* –*no*-*var* –*bnd_distance 100000* –*overlap 0.01 *>* query.vcf*

Here, input.vcf represent the VCF produced through the analysis of the various pipelines (TIDDIT, Sniffles, Longranger, Bionano Solve), and truthset.bedpe represent the bedpe file containing the *t*(X;21;19;4) BPJs.

A call was considered to represent a BPJ if both of its reported breakpoint positions were within 100 Kbp of the confirmed breakpoint. An SV call being close to multiple confirmed BPJs were considered to represent only one BPJ (the closest one). The distance of 100 Kbp was selected based on the size of the aberrant fragments, and to account for the lower resolution of Optical mapping.

### Hi-C data and TAD analysis

Hi-C data produced from cerebellar astrocytes were downloaded via the ENCODE experiment ENCSR011GNI website (https://www.encodeproject.org/experiments/ENCSR011GNI/). The analyses were based on the publicly available bed file of TAD regions (Lajoie et al. 2015). TAD regions were coupled with breakpoints using TABIX (Li 2011): in these analyses, the TAD bed file was used as a database that was queried using the coordinates of the BPs. Next, the TADs were searched for protein-coding genes, querying the RefSeq gene annotation (assembly GCF_000001405.25) gff files with the positions of the affected TADs.

## Electronic supplementary material

Below is the link to the electronic supplementary material.

Breakpoint junction sequences for the 137 junctions. The junction sequences were obtained either through the sequencing data, through breakpoint PCR, or both. Some junctions were only supported by whole-genome sequencing data on one side due to the large inserted sequences, and were either confirmed by breakpoint PCR or through “walking” along the inserted sequence until the sequences met the junction sequence from the other side of the junction (PDF 692 kb)

A GTEx multigene query heatmap. The heatmap illustrate the per tissue expression level of protein-coding genes affected by the t(X;21;19;4) (TIFF 283 kb)

De novo assembly of the t(X;21;19;4). Contiguity plots illustrating contigs larger than 2 Mbp. The chromosomes of the human genome are labeled based on their cytoband patterns, while the contigs are colored based on which chromosome they align. A) Nanopore assembly, B) Linked read assembly, C) Nanopore-Optical map hybrid, D) nanopore-Linked read-Optical map hybrid (TIFF 10229 kb)

A contig consisting of multiple aberrant fragments. The position and origin of the fragments are presented in Supplementary Table 1 (TIFF 387 kb)

Gametes possible through alternate and adjacent I segregation (JPEG 394 kb)

Breakpoints: All breakpoints detected on all of the involved chromosomes, with start and stop coordinates and possibly deleted nucleotides. Segments: All identified segments involved in the rearrangements with start and stop coordinates and segment size, as well as total amount of identified segments originating from each of the chromosomes involved. Junctions: Details of the junctions that were characterized with possible microhomology, insertions or other. Der(4), Der(19), Der(21), Der(X), Der(7) and Der(11): Details of the segments that consisted each derivative chromosome with their size and orientation (XLSX 94 kb)

Supplementary material 7 (XLSX 10 kb)

## Data Availability

The datasets generated and/or analyzed during the current study are not publicly available due the European GDPR law but are available from the corresponding author on reasonable request.
