# Supplementary figures and images for: Hybrid sequencing resolves two germline ultra-complex chromosomal rearrangements consisting of 137 breakpoint junctions in a single carrier
Source: Hum Genet. 2020 Dec 14;140(5):775–90. doi: 10.1007/s00439-020-02242-3 (PMC8052244; doi:10.1007/s00439-020-02242-3)

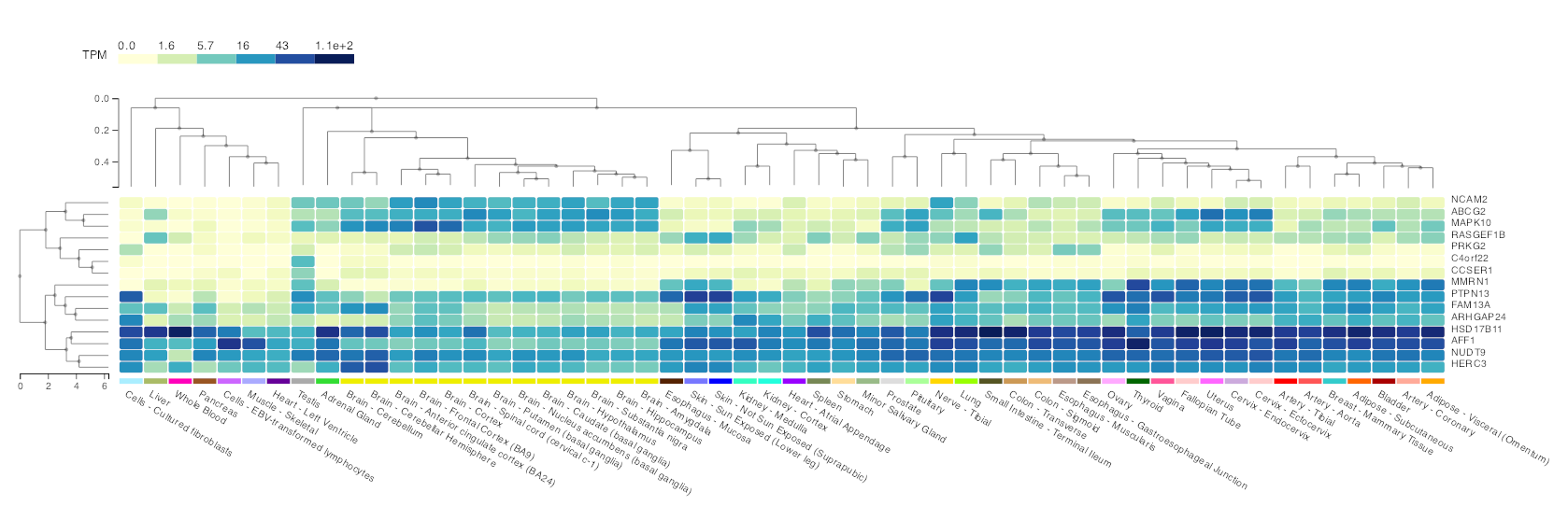

Supplement: Supplementary file 2 — A GTEx multigene query heatmap. The heatmap illustrate the per tissue expression level of protein-coding genes affected by the t(X;21;19;4) (TIFF 283 kb) [file 439_2020_2242_MOESM2_ESM.tiff]

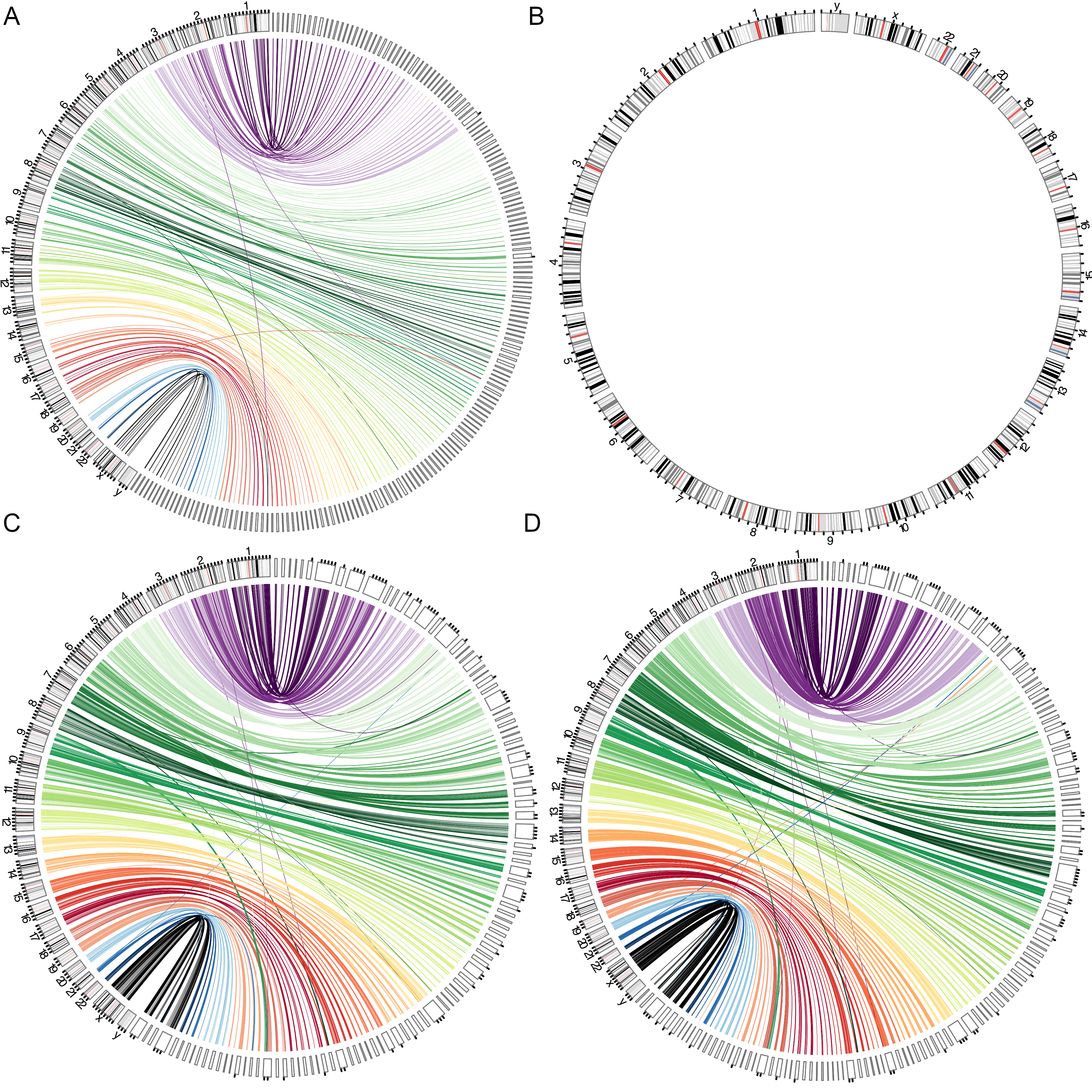

Supplement: Supplementary file 3 — De novo assembly of the t(X;21;19;4). Contiguity plots illustrating contigs larger than 2 Mbp. The chromosomes of the human genome are labeled based on their cytoband patterns, while the contigs are colored based on which chromosome they align. A) Nanopore assembly, B) Linked read assembly, C) Nanopore-Optical map hybrid, D) nanopore-Linked read-Optical map hybrid (TIFF 10229 kb) [file 439_2020_2242_MOESM3_ESM.tiff]

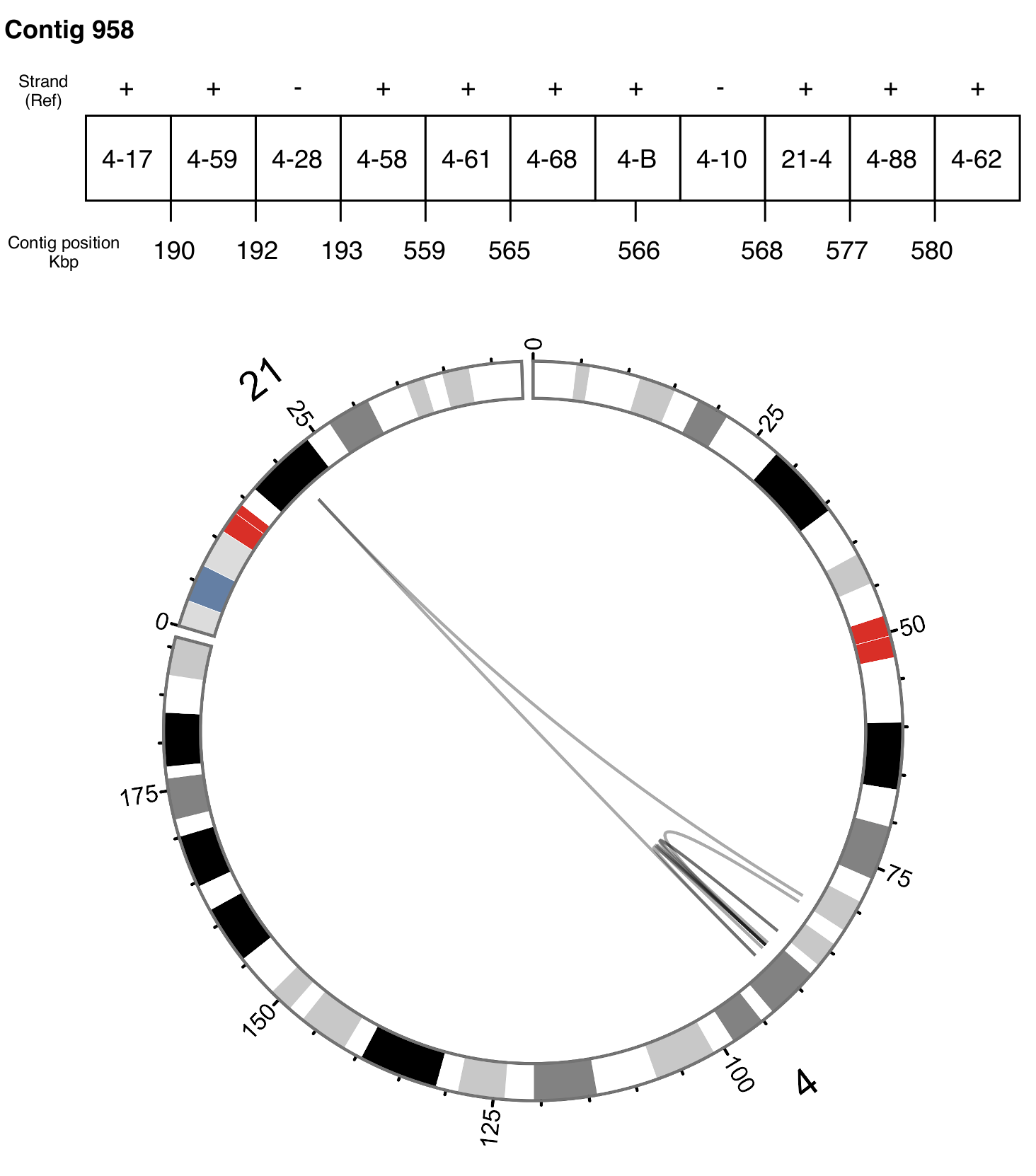

Supplement: Supplementary file 4 — A contig consisting of multiple aberrant fragments. The position and origin of the fragments are presented in Supplementary Table 1 (TIFF 387 kb) [file 439_2020_2242_MOESM4_ESM.tiff]

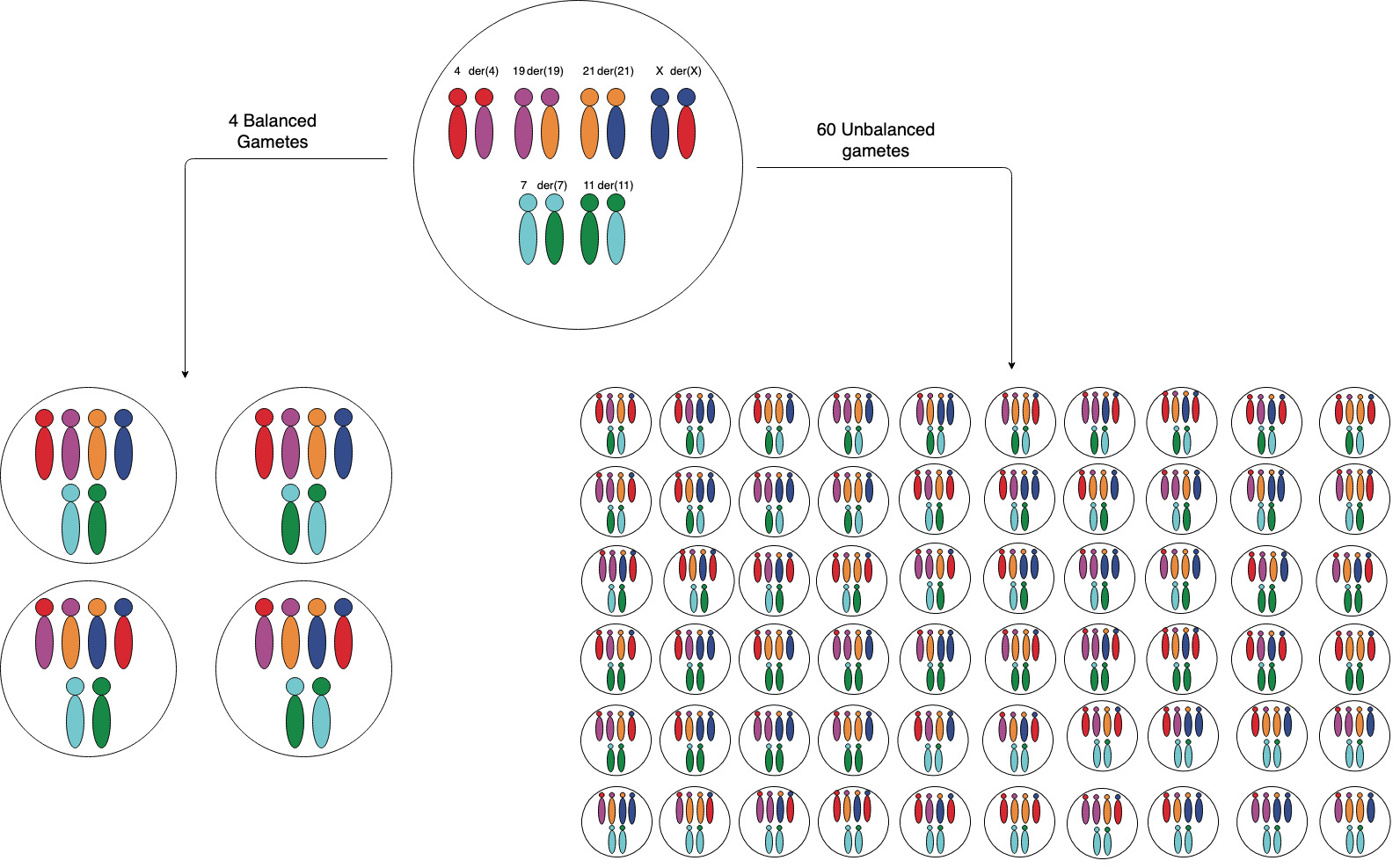

Supplement: Supplementary file 5 — Gametes possible through alternate and adjacent I segregation (JPEG 394 kb) [file 439_2020_2242_MOESM5_ESM.jpg]
