# Supplementary material for: Hybrid sequencing resolves two germline ultra-complex chromosomal rearrangements consisting of 137 breakpoint junctions in a single carrier
Source: Hum Genet. 2020 Dec 14;140(5):775–90. doi: 10.1007/s00439-020-02242-3 (PMC8052244; doi:10.1007/s00439-020-02242-3)
Supplement: Supplementary file 1 — Breakpoint junction sequences for the 137 junctions. The junction sequences were obtained either through the sequencing data, through breakpoint PCR, or both. Some junctions were only supported by whole-genome sequencing data on one side due to the large inserted sequences, and were either confirmed by breakpoint PCR or through “walking” along the inserted sequence until the sequences met the junction sequence from the other side of the junction (PDF 692 kb) [file 439_2020_2242_MOESM1_ESM.pdf]

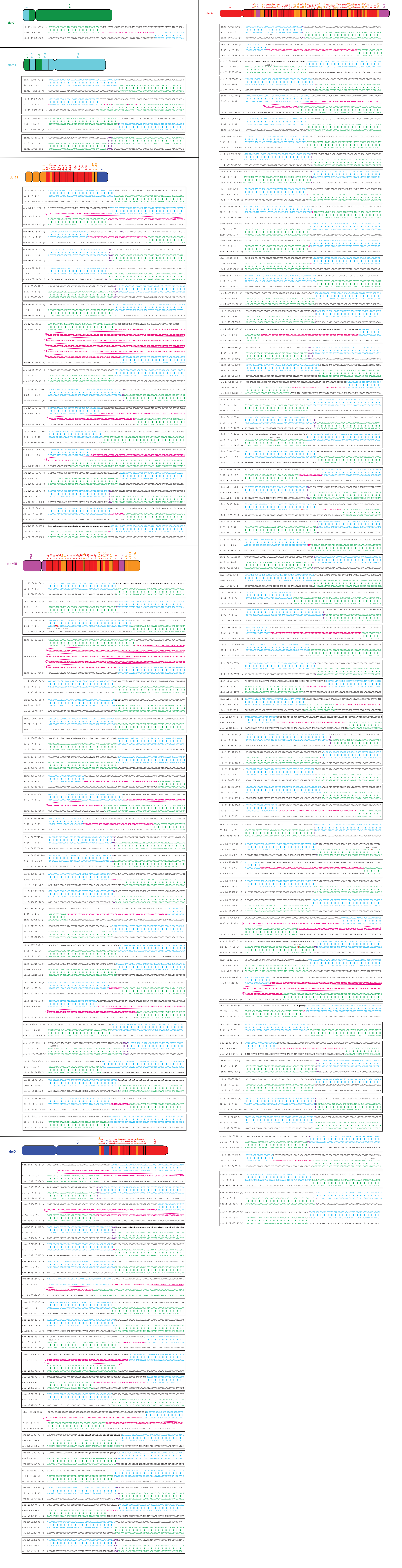
der7
der11
der21
der19
derX
der4
